# Dreams of the *Rarebit Fiend*: food and diet as instigators of bizarre and disturbing dreams

**DOI:** 10.3389/fpsyg.2015.00047

**Published:** 2015-02-17

**Authors:** Tore Nielsen, Russell A. Powell

**Affiliations:** ^1^Dream and Nightmare Laboratory, Center for Advanced Research in Sleep Medicine, Hôpital du Sacré-Coeur de Montreal, Department of Psychiatry, Université de MontrealMontreal, QC, Canada; ^2^Department of Psychology, Grant McEwan UniversityEdmonton, AB, Canada

**Keywords:** nightmares, dreaming, sleep, food, diet, fasting, internal awareness

## Abstract

In the early 1900s, the *Dream of the Rarebit Fiend* comic strip conveyed how the spicy cheese dish Welsh rarebit leads to bizarre and disturbing dreams. Today, the perception that foods disturb dreaming persists. But apart from case studies, some exploratory surveys, and a few lab studies on how hunger affects dreaming, there is little empirical evidence addressing this topic. The present study examines three aspects of the food/dreaming relationship; it attempts to: (1) assess the prevalence of the perception of food-dependent dreaming and the types of foods most commonly blamed; (2) determine if perceived food-dependent dreaming is associated with dietary, sleep or motivational factors; and (3) explore whether these factors, independent of food/dreaming perceptions, are associated with reports of vivid and disturbing dreams. Three hundred and ninety six students completed questionnaires evaluating sleep, dreams, and dietary habits and motivations. Items queried whether they had noticed if foods produced bizarre or disturbing dreams and if eating late at night influenced their dreams. The perception of food-dependent dreaming had a prevalence of 17.8%; with dairy products being the most frequently blamed food category (39–44%). Those who perceived food-dependent dreaming differed from others by reporting more frequent and disturbing dreams, poorer sleep, higher coffee intake, and lower Intuitive Eating Scale scores. Reports of disturbing dreams were associated with a pathological constellation of measures that includes poorer sleep, binge-eating, and eating for emotional reasons. Reports of vivid dreams were associated with measures indicative of wellness: better sleep, a healthier diet, and longer times between meals (fasting). Results clarify the relationship between food and dreaming and suggest four explanations for the perception of food-dependent dreaming: (1) food specific effects; (2) food-induced distress; (3) folklore influences, and (4) causal misattributions. Research and clinical implications are discussed.

## Introduction

**Figure 1:**
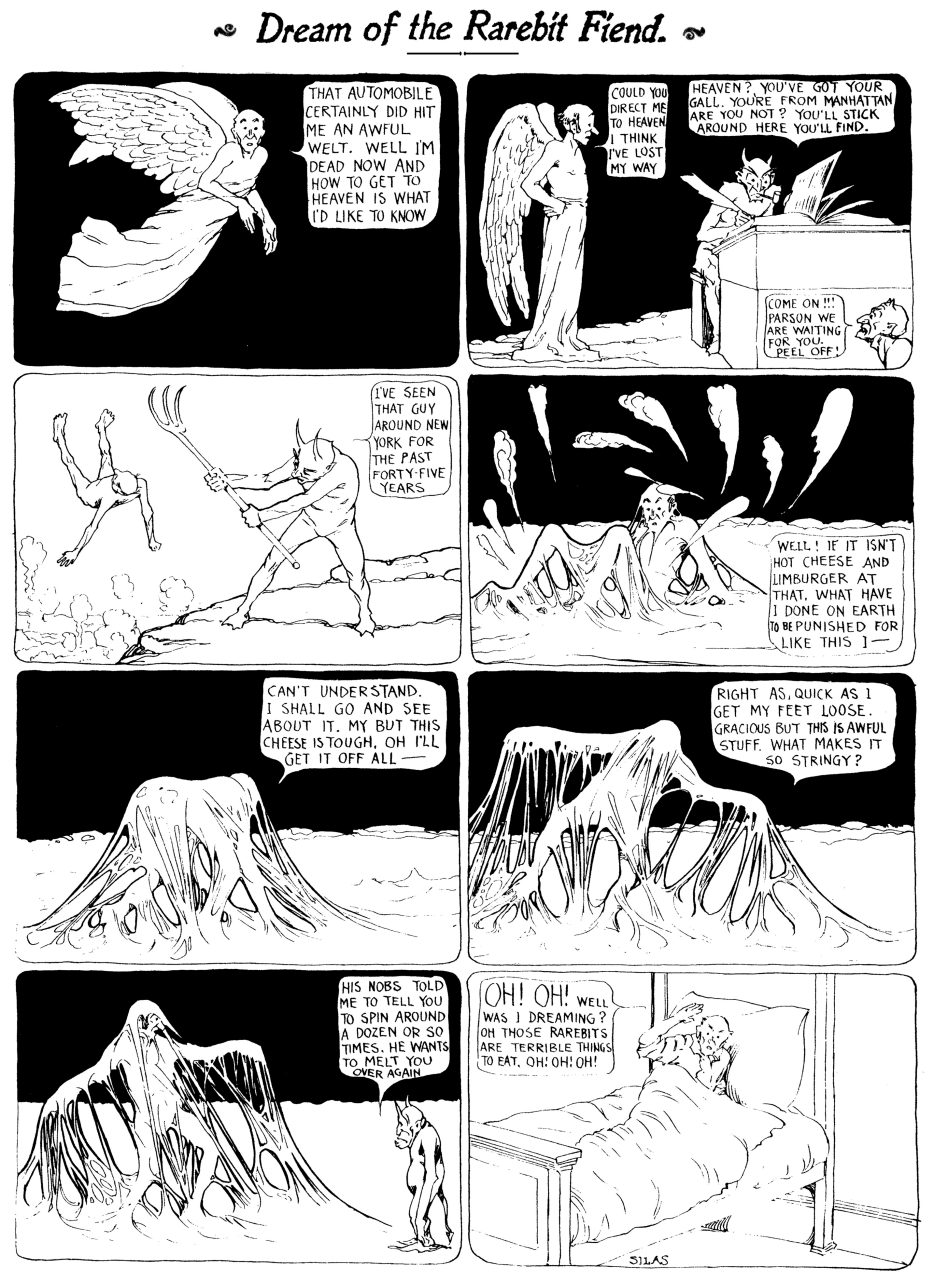
**Sample *Dream of the Rarebit Fiend* comic strip by Wilson McCay (aka Silas) that was popular in the early 1900's and influenced popular culture for many years.** The cartoons usually ended with the protagonist (who differed from cartoon to cartoon) regretting having eaten Welsh rarebit, a dish of melted spiced cheese on toast. In the present study, dairy products, including cheese, and spicy foods were most often named by participants as foods that induce disturbing dreams.

For millennia, people have believed that the foods they eat can influence their dreams. The *Dream of the Rarebit Fiend* comic strip (see Figure 1), penned by Winsor McCay and published in the *New York Herald* from 1904 to 1925, is a striking example of how such beliefs have been expressed repeatedly in popular culture. Even today, anecdotes are common about how a recurrent dream, bizarre dream, or nightmare was triggered by eating a particular food or by eating too much or too late at night. Yet there is surprisingly little empirical research that directly addresses the question of food-dependent dreaming. Although evidence exists that sleep may be facilitated or disrupted by different types of foods—for example, foods that are rich in tryptophan, caffeine or alcohol, or even specific foods such as milk, kiwis or tart cherry juice (see review in Peuhkuri et al., 2012)—the effects of food on dreaming remain largely in the realm of speculation. Further, common beliefs and traditions about which foods influence dreaming are not well-documented, even though such subjective impressions might provide useful starting points for more objective research on the topic. The present study takes a first step toward investigating this issue by exploring how people perceive food to influence their dreams and whether these perceptions are associated with sleep characteristics and dietary habits and motivations.

Evidence that food directly affects dreaming is scarce. We found, for example, a single correlational study of dietary preferences and dreaming (Kroth et al., 2007), conducted with 7 men and 42 women, which revealed that participants who expressed a preference for organic foods reported more frequent dream recall, recurring dreams and meaningful dreams, as well as more dreams containing particular themes such as flying, risk-taking, sex, and water. In contrast, participants who expressed a preference for fast foods reported less frequent dream recall, as well as fewer recurring dreams, nightmares, and sexual dreams. While the authors of this study rightly consider the findings preliminary, the small sample size (especially for males), lack of correction for multiple correlations, and other methodological weaknesses suggest caution in interpreting these findings.

We also found evidence of a single, industry-sponsored laboratory study on the effects of food on dreaming (British Cheese Board, 2005). In 2005, the British Cheese Board conducted a study with 200 volunteers on the effects of eating cheese on dreams. The study purportedly found no evidence for the notion that eating cheese prior to sleep can induce nightmares, but did find evidence that different types of cheese can induce different types of dreams—for example, eating Stilton cheese often led to crazy or vivid dreams while eating cheddar cheese often led to dreams of celebrities. But although the results were widely reported in the media [e.g., “*Sweet dreams are made of cheese*,” Anonymous (Daily Mail), 2005], they were never published in a peer-reviewed journal, and the available information about the study is extremely sparse.

If direct evidence about food-dependent dreaming is lacking, some indirect evidence can be gleaned from studies on the effects of hunger and thirst on dreaming, from how individuals dream about food, and from anecdotal information concerning people's beliefs about the effects of food on dreams. These three sources of evidence are considered below.

### Hunger and thirst influences on dreaming

Hunger in the form of fasting was a known trigger of vivid dreams and visions among indigenous peoples of North America (for review and examples see Lincoln, 1970) and among some early dream incubation cults such as the Oracles of Trophonius (Meier, 1967, p. 109) and Amphiaros (MacCulloch, 1912) in Greece, and in even earlier Egyptian dream temples such as the Imuthes temple in Memphis and the Thoth temple in Khimunu (Foucart, 1912). In many of these contexts, fasting formed part of a quest for visionary contact with the spirit world that involved several forms of privation and sacrifice, e.g., sleep deprivation, social isolation, exposure, or threats of animal attack. Causal relationships between fasting and dreams are not easy to discern from these historical and anthropological sources, but it was generally believed that the absence of food, leading to acute or chronic hunger, can trigger vivid, sometimes transformative dreams.

On a more prosaic level, hunger was apparently a trigger for a dream of Freud's daughter, Anna, when she was 19 months old (Freud, 1900). Her celebrated “strawberry dream” took place following a day in which she went without food after over-consuming strawberries and vomiting in the morning. In her sleep she called out “*Anna F(r)eud, St'awbewy, wild st'awbewy, om'lette, pap!*,” which Freud took to indicate that even this young child was capable of dreaming of the foods she hungered for. Freud considered the drives of hunger and thirst to constitute the sole somatic sources of very simple dreams of food and drink, the simplest of wish-fulfillment dreams as it were.

Some support for these ancient beliefs and psychoanalytic impressions was provided by O'Nell (1965), who examined relationships between chronic food and thirst frustration and the appearance of food and liquids in the dreams of different cultural groups. Dream reports were contributed by young males from four cultural groups that differed in “food frustration” scores. These scores were calculated on the basis of the presence and severity of fasting, under-nutrition, and malnutrition in their respective cultures. Evaluation of food and drink themes in the dreams of these four groups revealed a clear correspondence between degree of food frustration and dreams of food, i.e., higher food and thirst frustration was associated with more food and thirst dreams, respectively.

Following discovery of REM sleep and its link with vivid dreaming, further evidence was marshaled from laboratory settings that linked the motivational effects of hunger and thirst to dreaming (Dement and Wolpert, 1958; Bokert, 1968). Dement and Wolpert (1958) collected a total of 15 REM sleep dreams from three subjects who were deprived of fluids for 24 h prior to sleeping in the laboratory on each of five nights. Five of the dreams (33%) contained thirst-related content, but these did not depict the dreamer as thirsty or in the act of drinking. A more extensive study was conducted by Bokert (1968) involving 18 female nurses who were deprived of food and drink for the 8 h prior to coming to the sleep laboratory where they then ate a highly spiced meal. Prior to awakenings from REM sleep for dream collection, a recording of the phrase “a cool delicious drink of water” was played repeatedly. Using a detailed grid of drink-related words, dreams about liquids were seen to increase, with some subjects even dreaming about the stimulation phrase. Interestingly, those who had dreams of satisfying their thirst drank less upon awakening than did subjects who had dreams of simply being thirsty.

Together, historical accounts and some systematic studies support the notion that hunger and thirst may produce more vivid, bizarre, and personally significant dreams. The widespread belief in the intentional use of fasting to stimulate dreaming likely reflects this influence. Such studies are nonetheless limited in what they reveal about direct relationships between food and dreaming.

### Dreams about food

Clinicians have expressed an interest in dreams about food, frequently in relation to patient symptomatology. Hamburger (1958), for example, collected a large corpus of food and eating dreams from four female analytic patients, two of whom were normal weight with no eating problems, one of whom was obese, and another of whom had bulimia. Of 1928 total dreams collected by the patients, 229 (11.9%; range across patients: 10.6–18.1%) were judged to deal with themes of food, eating, and associated settings, such as kitchens, dining rooms and restaurants. In 3 of 4 patients, such dreams tended to diminish in frequency as psychotherapy progressed. In no cases did patients notice food dreams arising in response to feelings of hunger during the preceding day nor did they report waking up from these dreams feeling hungry. Hamburger concluded that food dreams constitute a typical theme that derives from latent “oral needs,” essentially substituting one type of gratification (sexual, dependency) for another (alimentary).

Several content studies provide some indication of the prevalence of food and drink themes in dreams. First, the prevalence of taste and smell in dreams is moderately low; 36.6 and 39.0% of participants retrospectively report having experienced any kind of gustatory or olfactory dream imagery, respectively, and prevalence estimates from 2 to 3-week home logs are even lower: 13.4 and 15.2%, respectively (Zadra et al., 1998). In the latter study, only 0.86 and 1.01%, respectively, of over 3300 dreams contained such imagery. Second, the Hall and van de Castle dream content norms (Hall and van de Castle, 1966) indicate that food as an object occurred in the dreams of only 1.4% of male and 1.0% of female college students (1.2% overall), and the broader theme of orality (including *eating/drinking, being in a place of eating/drinking, preparing or securing food/drink*) was also relatively infrequent, occurring in the dreams of only 16.0% of males and 17.0% of females. Third, the typical dream theme of “eating delicious foods” has a lifetime prevalence among University students of 30.7% (Nielsen et al., 2003). A similar value was found in a larger sample of internet respondents: 26.9% (4307 of 16,030) dreamed this theme at least once, including 23.0% (848 of 3681) of males and 28.0% (3459 of 12,349) of females (χ^2^_(1)_ = 35.69, *p* < 0.000001; unpublished). Among eating disordered patients whose dreams were sampled in a single laboratory night, dreams of food were much more prevalent. One study found them in 58% of patients with bulimia, 26% of patients with anorexia, and 44% of patients with anorexia and bulimia combined (Dippel et al., 1987). Similarly, patients with a diagnosis of migraine headache were retrospectively more likely to report taste (31%) and smell (36%) in their dreams than were control subjects (20 and 20%, respectively) (Lovati et al., 2014).

In sum, the few available studies of dreams about food reveal a relatively low prevalence of dreams of eating *per se*, with the possible exception of individuals with eating disorders and other health conditions, but a higher prevalence of dreams about orality and food associations. While limited, such findings nonetheless demonstrate that dreaming about food and eating is not uncommon and also suggest that the effects of food on dreaming may be associated with health and personality factors (see also review by Strickler, 2005).

### Beliefs about the effects of food on dreams

Beliefs that dreams can be influenced by food are widespread and often circulated—and recirculated—by the media. Dream researchers are frequently asked about food effects on dreams, often in advance of food-centric holidays such as Halloween (candy), Easter (chocolate), or Christmas (turkey, general gluttony). An often-mentioned notion is the classic “Pickled Walnut” theory, of unknown origin, which stipulates that eating spicy or unusual foods before sleep will lead to bizarre dreams or nightmares. These beliefs have also had currency in common medical opinion. For example, the famous psychoanalyst, Ernest Jones, in his influential treatise, *On the Nightmare* (1931), noted that “in the current medical view they [nightmares] are ascribed to physical disturbances in the alimentary, respiratory or circulatory system, disturbances of either an irritating nature, such as undigested food, or of a mechanical nature, such as a distended stomach” (p. 344). He also named cucumbers as “the article of food that is most looked askance at in relation to Nightmare” (p. 38). Similar sentiments concerning the relationship of food to nightmares have been voiced by medical practitioners since antiquity; Hippocrates (1931), for example, stated that “monstrous bodies that are seen in sleep and frighten a man indicate [among other possibilities] a surfeit of unaccustomed food.…” (p. 443).

Perhaps not surprisingly then, beliefs about the effect of food on dreams have remained current among the general public. This was strikingly reflected in the recurring food themes of the previously mentioned comic strip, *Dream of the Rarebit Fiend*, from the early 1900s. Protagonists in these cartoons had bizarre or disturbing dreams that they blamed on their pre-sleep meals of Welsh rarebit (a dish of melted spiced cheese on toast) or, less commonly, mince pie. A similar literary reference is that of Ebenezer Scrooge attributing one of his ghostly visions to “*an undigested bit of beef, a blot of mustard, a crumb of cheese, a fragment of an underdone potato*” in Charles Dickens' *A Christmas Carol* (1843). One journalist's introduction to a piece about food and dreams in *New Scientist* magazine (Jones, 2000) sums up this widespread cultural belief as follows:

We've all heard the story a thousand times: eating too much food just before bed can give you bad dreams. Especially if it's spicy, or fermented, and definitely if it's cheese that smells like your sock drawer. I've noticed it. My mom's warned me about it. It's established fact, isn't it?

A prevailing belief in the power of cheese to induce bad dreams appears to have also been the motivation behind the aforementioned unpublished study sponsored by the British Cheese Board (2005): “‘Now that our Cheese and Dreams study has finally debunked the myth that cheese gives you nightmares we hope that people will think more positively about eating cheese before bed,’ says Nigel White, British Cheese Board secretary.”

Although the belief that foods can affect one's dreams appears to be prevalent, little empirical evidence has been collected on the issue. An early survey by Laird (1920) assessed 100 participants' beliefs about dreaming, hypnosis, telepathy, and the subconscious. Fifteen participants (15%) indicated that a cause of dreaming was the kind or amount of food eaten. This was nonetheless a majority of the 28% of participants who gave a positive answer to this question (other causes mentioned were “worry,” “conscience,” and “disturbed sleep”).

In sum, a sampling of medical and popular culture references, along with an early survey study, indicates that belief in the ability of food to affect dreams—especially in a negative fashion—has been and continues to be common. The present study examines beliefs about food-dependent dreaming by asking undergraduate students whether and how foods can affect their dreams and which foods are most influential. The study also assesses whether participants who did and did not report experiencing food-dependent dreaming differed on measures of sleep, dreams, and diet-related habits and motivations, as well as the relationship of these same variables to reports of vivid and disturbing dreams independent of the perception of food-dependent dreaming.

## Materials and methods

### Participants

Participants were 396 undergraduate students—130 males (*M*_age_: 21.6 ± 5.28 years), 264 females (*M*_age_: 21.4 ± 5.10 years; *t*_392_ = 0.37, *p* = 0.715), and two for whom age and sex were unspecified—enrolled in first-year psychology courses at a Canadian university. The study was approved by the university's Research Ethics Board. Each participant provided informed consent and received partial course credit for their participation. Responses from all 396 respondents were entered into correlational analyses. However, 14 participants failed to respond to one of the food-related dreaming items, leaving a total of 382 participants for the group comparisons: 126 males (*M*_age_: 21.6 ± 5.33 years), 255 females (*M*_age_: 21.4 ± 5.15 years; *t*_379_ = 0.39, *p* = 0.695), and one sex unspecified.

### Procedures

The questionnaires used in this study formed part of a larger on-line survey assessing food consumption patterns and various aspects of behavioral and psychological functioning. Following the survey, participants read a thorough debriefing that explained the nature and importance of the study. The email address of the primary researcher was also provided for students who wanted more information. The survey measures used in the present study are described below.

#### Sleep Quality Scale (SQS)

This 28-item questionnaire (Yi et al., 2006) assesses problems in sleep quality over the past month, including daytime dysfunction due to sleepiness, restoration from sleep, difficulties in waking or falling asleep, sleep satisfaction, and awakenings during sleep. Items are rated on a 4-point scale with endpoints labeled 1-*never or rarely* (up to 1–3 times a month); 2-*sometimes* (1–2 times a week); 3-*often* (3–5 times a week) and 4-*almost always* (6–7 times a week). The SQS has been shown with college students to have strong internal consistency and test-retest reliability, as well as strong concurrent and construct validity (Yi et al., 2006). In the present study, we used four of the SQS subscales: (1) daytime dysfunction due to sleepiness; (2) difficulty falling asleep; (3) sleep dissatisfaction; and (4) sleep maintenance problems. We also added an in-house measure of sleep time, i.e., the average number of hours slept per day. (All in-house scales and items used in this study are presented in the Appendix).

#### Three Factor Eating Questionnaire (TFEQ; Stunkard and Messick, 1985)

The TFEQ is a 51-item self-report questionnaire that assesses eating-related thoughts and behaviors with three subscales (*cognitive restraint, disinhibition, hunger*). However, because the TFEQ factor structure has been questioned (Karlsson et al., 2000), we calculated Karlsson et al.'s more psychometrically sound TFEQ 18-item revised (TFEQ-18R) questionnaire from participants' raw scores. The three subscales of the TFEQ-18R include: *cognitive restraint* (CR; test-retest *r* = 0.81–0.93; Cronbach's alpha = 0.93), or the propensity to control eating to influence body weight and body shape; *uncontrolled eating* (UE; *r* = 0.80–0.86; CA = 0.91), or the propensity to lose control over eating when feeling hungry or when exposed to external stimuli; and *emotional eating* (EE; *r* = 0.75–0.83; CA = 0.85), or the tendency to overeat in response to dysphoric mood states, e.g., when feeling lonely, anxious, or depressed.

#### Intuitive Eating Scale (IES)

This 21-item instrument (Tylka, 2006) measures an individual's propensity to utilize physical hunger/satiety cues, rather than situational and emotional cues, when deciding what, when and how much to eat. It consists of three subscales: *unconditional permission to eat* (CA = 0.89), *eating for physical reasons* (CA = 0.86), and *reliance on hunger/satiety cues* (CA = 0.72). Only the latter subscale was used in the present study. IES scores, including reliance on hunger/satiety cues, are associated positively with psychological well-being and negatively with eating disorder symptoms, body dissatisfaction, poor interoceptive awareness, pressure for thinness, and internalization of the thin-ideal stereotype (Tylka, 2006).

#### Diet quality questionnaire

This in-house questionnaire was designed to estimate the quality of participants' diets by having them retrospectively report the extent to which they ate foods that are prototypically considered to be “healthy” or “unhealthy,” as well as the amount of coffee they drank. The questionnaire items were modified versions of scale items used by Thompson et al. (2000). Four items (green salads, vegetables, fruit, whole grains) were averaged to form a *healthy foods* variable, and two items (non-diet soft drinks, low-nutrient snacks) were averaged to form an *unhealthy foods* variable.

#### Eating Behavior Questionnaire

This in-house questionnaire assessed select attributes of eating behavior that could potentially influence sleep and dreams. One item asked if the participant was currently following a calorie-reducing diet; another assessed the longest time that elapsed between eating or drinking during a typical day—which we viewed as a proxy measure of short-term fasting. A third item, drawn from the *Eating Disorders Diagnostic Scale* (Stice et al., 2000), assessed the tendency to engage in binge-eating behavior.

#### Perceived Food-Dependent Dreaming (FDD) Questionnaire

This in-house questionnaire assessed the perceived effects of food on dreaming with three binary (yes/no) and three open-ended questions. Two binary questions asked whether participants had ever noticed foods that led to disturbing dreams or bizarre dreams while a third asked whether they had ever noticed if eating late at night had affected their sleep or dreams. [Dreams that are either disturbing or bizarre may overlap to the extent that both can involve negative emotion. But the two are distinct in that bizarre dreams, which contain impossible (e.g., flying) or improbable (e.g., winning a lottery) events, are not necessarily negative]. For participants who answered “yes” to each of the three binary questions, the following open-ended question asked participants to give specific examples. From the eating late at night items, we calculated a new variable specific to dreaming that was scored 1 if participants mentioned an effect on dreams (rather than sleep) in response to the open-ended question and was scored 0 otherwise. We then created a perceived food-dependent dreaming (FDD) variable in which participants who reported either that eating a particular food led to disturbing or bizarre dreams or that eating late at night affected their dreams were given a score of 1 (FDD+) and those who did not were given a score of 0 (FDD−).

#### Dream Characteristics Questionnaire

Disturbing and normal dreams over the past 2 weeks were assessed with a 7-item in-house questionnaire that assessed three aspects of disturbing dreams (recall frequency, distress, #wake-ups) and four aspects of non-disturbing dreams (recall frequency, clarity, realism, color). To reduce the number of variables for subsequent correlational analyses, responses to the seven questions were first subjected to a principal components factor analysis with varimax rotation (Kaiser normalization). A clear two-factor solution accounting for 58.2% of the variance emerged which consisted of a *disturbing dreams* factor (%variance = 34.7) that contained all three disturbing dreams items (component loadings: DD-distress = 0.865; DD-wakeUp = 0.852; DD-freq = 0.752), and a *vivid dreams* factor (%variance = 23.5) that contained the remaining four items (DR-realism = 0.777; DR-clarity = 0.732; DR-freq = 0.677; DR-color = 0.613). *Disturbing dreams* and *vivid dreams* factor scores were used as primary endpoints for subsequent analyses.

### Statistical analyses

Distributions of participants as a function of sex and responses to the three FDD items were evaluated with χ^2^ analyses. FDD group differences on sleep, dreaming, and other variables were compared using a series of MANCOVAs with FDD group (FDD+, FDD−) as between groups factors and the multivariable groupings of dependent measures shown in **Table 3**. Effects were subsequently assessed with univariate statistics and *post-hoc t*-tests. Significance threshold was set at *p* < 0.05. To assess whether dreams and food/diet measures were associated independent of FDD status, partial correlations were conducted while covarying FDD status. Because of strong correlations observed between disturbed sleep and other measures, partial correlations that covaried relevant sleep disturbance scores were also conducted.

## Results

### Descriptive

As shown in Table 1, 68 of 382 (17.8%) participants indicated that their dreams were affected either by eating particular foods (11.5%) or eating late at night (9.5%). Food-dependencies were observed in approximately equal proportions for disturbing dreams (8.9%) and bizarre dreams (7.6%). Distributions of food-dependencies for males and females did not differ for disturbing dreams (χ^2^ = 0.735, *p* = 0.391), bizarre dreams (χ^2^ = 0.414, *p* = 0.520), disturbing and bizarre dreams combined (χ^2^ = 0.436, *p* = 0.606), or dreams affected by either foods or eating late (χ^2^ = 0.165, *p* = 0.684). The two sexes were therefore combined for all subsequent analyses.

**Table 1:** **Participants perceiving their bizarre or disturbing dreams to be influenced by food or by eating late at night**.

**Item**^†^	**Males**		**Females**		**All**		**Total**
	**(*N = 126*)**		**(*N = 255*)**		**(*N = 382*)**		
	**Yes**	**No**	**Yes**	**No**	**Yes**	**No**	
	***N* (%)**	***N* (%)**	***N* (%)**	***N* (%)**	***N* (%)**	***N* (%)**	
1. Food causes disturbing dreams^a^	9 (7.1)	117 (92.9)	25 (9.8)	230 (90.2)	34 (8.9)	347 (91.1)	381
2. Food causes bizarre dreams^b^	8 (6.3)	118 (93.7)	21 (8.2)	235 (91.8)	29 (7.6)	353 (92.4)	382
3. Eating late affects sleep or dreams^c^	29 (23.2)	96 (76.8)	71 (28.0)	183 (72.0)	100 (26.4)	279 (73.6)	379
4. Eating late affects dreams^d^	12 (9.6)	113 (90.4)	24 (9.4)	230 (90.6)	36 (9.5)	343 (90.5)	379
5. 1 or 2 (Food causes bizarre or disturbing dreams)	13 (10.3)	113 (89.7)	31 (12.1)	225 (87.9)	44 (11.5)	338 (88.5)	382
6. 1 or 2 or 4 (FDD+: above item plus eating late affects dreams)	21 (16.7)	105 (83.3)	47 (18.4)	209 (81.6)	68 (17.8)	314 (82.2)	382

† One participant failed to complete item 1; three participants failed to complete items 3 and 4.

a Have you ever noticed certain foods that seemed to lead you to have a disturbing dream? (no/yes).

b Have you ever noticed certain foods that seemed to lead you to have a bizarre dream? (no/yes).

c Have you ever noticed that eating late at night seems to affect your dreams or your sleep? (no/yes)

d Dream-related comment given to “In what way does eating late at night affect your dreams or your sleep?” (no/yes).

**Figure 2:**
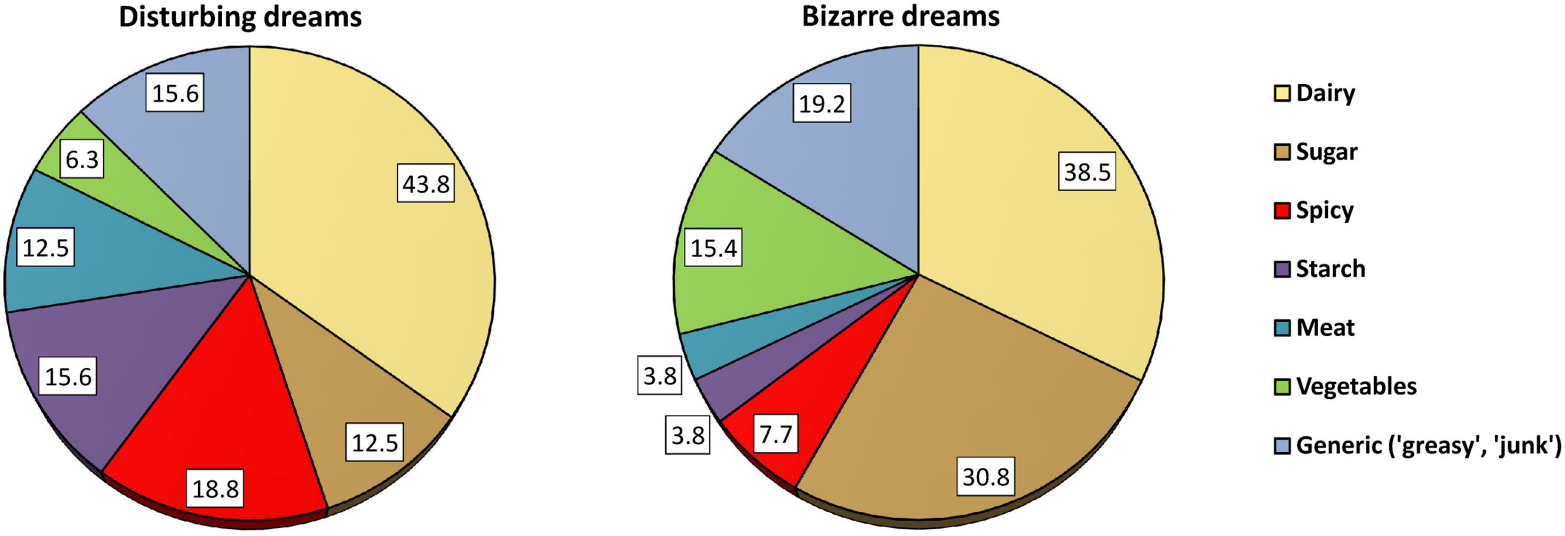
**Proportions of participants who identified specific food types as influencing disturbing and bizarre dreams**.

Table 2 lists the categories and frequencies of foods that were given in response to the open-ended questions asking for details about foods that influenced disturbing and bizarre dreams. The most frequent category mentioned (see Figure 2) was dairy, and included cheese, milk, hot milk, ice cream, yogurt, pizza, and poutine. Other foods typically containing cheese (e.g., pasta) were excluded from this category only because they also contained a predominance of other ingredients. It was also unclear from participants' mentions of “greasy” foods whether dairy was intended. Sweets and chocolate were the second most frequently mentioned category of dream-influencing foods, although these were mentioned more than twice as often for bizarre dreams (30.8%) than for disturbing dreams (12.5%; χ^2^ = 2.92, *p* = 0.088). The same was true for vegetables, although these were only infrequently mentioned. Conversely, spicy and starchy foods and meat were all mentioned more than twice as often for leading to disturbing dreams as they were for leading to bizarre dreams.

**Table 2:** **Foods identified as influencing disturbing and bizarre dreams**.

	**Disturbing dreams (*N* = 32)**		**Bizarre dreams (*N* = 26)**	
	***N***	**%**	***N***	**%**
**Dairy products**	**14**	**43.8**	**10**	**38.5**
Cheese	4	12.5	2	7.7
Any dairy	4	12.5	4	15.4
Milk	2	6.3	1	3.8
Pizza	3	9.4	3	11.5
Poutine	1	3.1		
**Sugar**	**4**	**12.5**	**8**	**30.8**
Sugar, sweets, candy	2	6.3	5	19.2
Chocolate	2	6.3	3	11.5
**Spicy**	**6**	**18.8**	**2**	**7.7**
Any spicy food	3	9.4	1	3.8
Pickles	3	9.4	1	3.8
**Starch**	**5**	**15.6**	**1**	**3.8**
Pasta	4	12.5		
Noodles	1	3.1	1	3.8
**Meat** (inc. steak, hamburger, hot dog)	**4**	**12.5**	**1**	**3.8**
**Vegetables** (beans, peanut butter, carrots, mushrooms)	**2**	**6.3**	**4**	**15.4**
**Generic**	**5**	**15.6**	**5**	**19.2**
Junk, fast foods	2	6.3	3	11.5
Greasy or deep-fried	3	9.4	2	7.7

Because some participants identified more than one food type, the totals in each column exceed 100%.

The probability of observing this proportion of dairy-related foods being named as responsible for either disturbing or bizarre dreams was calculated with binomial tests using the expected proportion of a food type calculated as the likelihood of observing any of the 7 food types being named equally often, or 1/7 = 14.28%. Thus, for disturbing dreams, the probability of observing 14 out of 32 (43.8%) instances of dairy-related foods was *Z* = 4.762, *p* < 0.0001. No other food type was statistically significant. For bizarre dreams, the probability of observing 10 out of 26 (38.5%) instances of dairy-related foods was *Z* = 3.527, *p* = 0.0004 while the probability of observing 8 out of 26 (30.8%) instances of sugar-related foods was *Z* = 2.245, *p* = 0.025. No other food types were significant.

The open-ended question about whether eating late influenced sleep and dreams produced a total of 89 responses; of these, 36 (40.4%) specifically mentioned changes in dreaming. A majority of these respondents, 47.2%, indicated that eating late brought on nightmares or disturbing dreams; 22.2% indicated that eating late made their dreams more bizarre, odd, strange, or crazy; 19.4% that they became more vivid, realistic or creative; 13.9% that they became more nonsensical or meaningless; 8.3% that they became more abundant; and 8.3% that they related more closely to the previous day's experiences.

### Questionnaire correlations

Pearson correlations between all dependent measures of the study are shown in Table 3. Measures are grouped by the multivariables contrasted in the group comparisons in the following section.

**Table 3:** **Pearson correlations for all participants between all study variables, grouped as for multi-variables in Table 4**.

	**1**	**2**	**3**	**4**	**5**	**6**	**7**	**8**	**9**	**10**	**11**	**12**	**13**	**14**	**15**	**16**	**17**
Disturbing dreams 1	–	0.8701	**0.0000**	**0.0000**	**0.0000**	**0.0000**	0.4607	0.5577	0.9292	0.0579	**0.0005**	0.8585	**0.0000**	**0.0026**	**0.0017**	0.0112	**0.0018**
Vivid dreams 2	−0.009	–	0.0212	0.3584	0.0228	0.4141	0.0293	**0.0045**	**0.0036**	0.4534	0.4403	**0.0005**	0.1453	0.1018	**0.0039**	0.0933	0.1264
SQS: Daytime dysfunction 3	**0.349**	−0.120	–	**0.0000**	**0.0000**	**0.0000**	**0.0000**	0.9601	0.0190	**0.0004**	**0.0000**	0.1076	**0.0000**	0.4870	**0.0000**	**0.0000**	**0.0000**
SQS: Difficulty falling asleep 4	**0.322**	−0.048	**0.522**	–	**0.0000**	**0.0000**	**0.0000**	0.0564	0.5533	0.0357	**0.0004**	0.3296	**0.0073**	0.0807	0.1386	0.0829	0.0192
SQS: Sleep dissatisfaction 5	**0.250**	−0.119	**0.569**	**0.421**	–	**0.0000**	**0.0000**	0.0180	0.3829	0.1671	0.0490	0.8151	**0.0034**	0.5133	**0.0073**	**0.0031**	**0.0002**
SQS: Sleep maintenance 6	**0.293**	0.043	**0.320**	**0.498**	**0.274**	–	**0.0044**	0.3769	0.5854	**0.0010**	**0.0041**	0.4240	0.2291	0.0428	0.2797	0.0316	0.3650
Hours of sleep 7	−0.039	0.114	**−0.315**	**−0.247**	**−0.385**	**−0.145**	–	0.8423	0.7445	**0.0065**	0.0496	0.7556	0.6538	0.9926	0.6324	0.0983	0.0109
Healthy foods 8	0.031	**0.148**	0.003	−0.098	−0.121	−0.045	0.010	–	**0.0000**	0.3930	0.0401	0.0480	0.4468	**0.0007**	0.7873	0.8244	0.3234
Unhealthy foods 9	0.005	**−0.152**	0.120	0.031	0.045	0.028	−0.017	**−0.275**	–	0.2599	0.3213	0.0153	0.1711	**0.0000**	0.0220	0.9061	0.2076
Coffee 10	0.099	0.039	**0.182**	0.108	0.071	**0.167**	**−0.139**	0.044	0.058	–	0.0377	0.5554	0.2102	0.4506	0.2032	**0.0091**	**0.0002**
Currently on diet 11	**−0.181**	−0.040	**−0.246**	**−0.182**	−0.101	**−0.146**	0.101	−0.105	0.041	−0.106	–	0.8396	**0.0000**	**0.0000**	**0.0002**	**0.0014**	**0.0000**
Time between meals 12	−0.009	**0.181**	−0.083	−0.050	−0.012	−0.041	0.016	−0.102	−0.125	−0.030	0.010	–	0.0189	0.0371	**0.0000**	0.0194	0.0225
Binge eating 13	**0.213**	−0.076	**0.247**	**0.137**	**0.149**	0.062	−0.023	−0.039	0.070	0.064	**−0.230**	−0.120	–	0.5954	**0.0000**	**0.0000**	**0.0000**
TFEQ: Cogn restraint 14	**0.156**	0.085	0.035	0.088	0.033	0.102	−0.001	**0.172**	**−0.337**	0.039	**−0.324**	0.107	0.027	–	**0.0011**	0.0768	0.2447
TFEQ: Uncontrolled eating 15	**0.163**	**−0.150**	**0.272**	0.075	**0.135**	0.055	0.025	0.014	0.117	0.065	**−0.187**	**−0.231**	**0.475**	**−0.164**	–	**0.0000**	**0.0000**
TFEQ: Emotional eating 16	0.132	−0.088	**0.205**	0.088	**0.149**	0.109	−0.085	−0.011	−0.006	**0.133**	**−0.163**	−0.120	**0.323**	0.089	**0.405**	–	**0.0000**
IES: Hunger/satiety cues 17	**−0.162**	0.080	**−0.244**	−0.119	**−0.186**	−0.046	0.130	0.050	−0.065	**−0.187**	**0.210**	0.117	**−0.387**	−0.059	**−0.305**	**−0.214**	–

Lower left triangle of table: Pearson correlations (bold coefficients are significant at p < 0.01); upper right triangle: exact p-values. Because some participants did not give valid responses for some measures, Ns vary between 357 and 396.

### Food-dependent dreaming group comparisons

FDD status was used to compare participants who did and did not perceive food to influence their dreams. FDD+ and FDD− groups differed significantly in age (*t*_380_ = 3.82, *p* = 0.004) with FDD+ participants being about 2.5 years older (*M* = 23.6 ± 7.0 years) than FDD− participants (21.0 ± 4.6 years) on average. Due to this Age discrepancy, Age was examined as a covariate in subsequent comparisons.

Results for multivariate assessments with Age as a covariate are shown in Table 4. Three of the five multivariate effects were significant (*p* < 0.05): **Dreams/Nightmares** (*T* = 0.020; *F*_2,363_ = 3.69, *p* = 0.026), **Sleep Disturbance** (Hotelling's *T* = 0.043; *F*_5, 371_ = 3.19, *p* = 0.008), and **Eating Motivation** (*T* = 0.026; *F*_4, 375_ = 2.42, *p* = 0.048). The **Dreams/Nightmares** multivariate main effect was due to the FDD+ group having greater scores on the *disturbing dreams* factor (*F*_1, 364_ = 5.35, *p* = 0.020). The **Sleep Disturbance** effect was due primarily to the FDD+ group having greater *difficulty falling asleep* (*F*_1, 375_ = 11.22, *p* = 0.001) and fewer *hours of sleep per day* (*F*_1, 375_ = 7.11, *p* = 0.008), but also to greater *daytime dysfunction* (*F*_1, 375_ = 4.41, *p* = 0.036) and greater *sleep dissatisfaction* (*F*_1, 375_ = 4.42, *p* = 0.036). A single univariate difference from the **Diet Quality** multivariable was that FDD+ participants reported drinking more *coffee* than did FDD− participants (*F*_1, 373_ = 5.73, *p* = 0.017). Finally, the multivariate **Eating Motivation** effect was due primarily to FDD+ participants scoring lower than FDD− participants on IES *reliance on hunger/satiety cues* (*F*_1, 378_ = 8.16, *p* = 0.005), but they also scored marginally higher on TFEQ-18R *emotional eating* (*F*_1, 378_ = 3.10, *p* = 0.079).

**Table 4:** **Mean (±SD) scores, multivariate and univariate main effects for participants saying “yes” (FDD+) or “no” (FDD−) to items querying if particular foods or eating late at night affected their dreams**.

**Item**	**FDD+ (SD)**	**FDD−^a^ (SD)**	**Group^b^ (FDD comparison with age covaried)**		**Group^c^ (FDD comparison with age and late eating covaried)**	
			***F*^d^**	***p***	***F*^d^**	***p***
**Multi-variable 1. Dreams/nightmares**			**3.69**	0.026^*^	**1.00**	**0.369**
Disturbing dreams	0.25 ± 1.00	−0.06 ± 0.99	5.35	0.020^*^	1.57	0.211
Vivid dreams	0.09 ± 0.97	−0.02 ± 1.02	1.84	0.176	0.41	0.524
**Multi-variable 2. Sleep Disturbance**			**3.19**	0.008^**^	**2.52**	0.029^*^
SQS: daytime dysfunction	1.89 ± 0.70	1.73 ± 0.55	4.41	0.036^*^	4.13	0.043^*^
SQS: difficulty falling asleep	2.06 ± 0.78	1.74 ± 0.69	11.22	0.001^***^	8.55	0.004^**^
SQS: sleep dissatisfaction	2.85 ± 0.86	2.62 ± 0.79	4.42	0.036^*^	0.32	0.571
SQS: sleep maintenance	2.13 ± 0.77	2.01 ± 0.84	0.61	0.437	0.02	0.898
Hours of sleep	6.81 ± 1.26	7.36 ± 1.29	7.11	0.008 ^**^	1.24	0.267
**Multi-variable 3. Diet Quality**			**1.93**	**0.124**	**0.78**	**0.507**
Healthy foods	5.10 ± 1.20	5.10 ± 1.38	0.03	0.861	0.00	0.982
Unhealthy foods	3.15 ± 1.50	3.20 ± 1.43	0.03	0.869	0.49	0.486
Regular coffee	4.05 ± 2.56	3.12 ± 2.21	5.73	0.017^*^	1.66	0.199
**Multi-variable 4. Eating Behavior**			**0.28**	**0.843**	**1.03**	**0.380**
Currently on diet^e^	1.85 ± 0.36	1.82 ± 0.38	0.50	0.478	0.75	0.387
Time between meals	3.63 ± 1.11	3.72 ± 1.04	0.27	0.607	1.66	0.198
Binge-eating	2.87 ± 1.67	2.92 ± 1.78	0.12	0.732	0.21	0.646
**Multi-variable 5. Eating motivation**			**2.42**	0.048^*^	**1.96**	**0.105**
TFEQ-18R: Cognitive restraint^f^	1.89 ± 0.47	1.88 ± 0.44	0.45	0.504	0.44	0.509
TFEQ-18R: Uncontrolled eating^f^	1.64 ± 0.36	1.58 ± 0.33	1.86	0.174	1.21	0.272
TFEQ-18R: Emotional eating^f^	1.47 ± 0.42	1.35 ± 0.39	3.10	0.079	5.82	0.016^*^
IES:Hunger/satiety cues^g^	3.47 ± 0.69	3.69 ± 0.56	8.16	0.005^**^	3.38	0.067

F and p scores are shown for age-covaried main effects for FDD comparisons as well as for FDD comparisons with late eating effect also covaried.

a FDD+/FDD−, participants reported “yes” or “no”, respectively to food-dependent dreaming (dreams were influenced by particular foods or by eating late).

b FDD+.

c FDD+ cohort while covarying perception of dreams being affected by “eating late”.

d Multivariate F- and p-values are shown for rows in bold font, univariate statistics for all other rows.

e 1, yes; 2, no.

f TFEQ−18R, Three-factor Eating Questionnaire 18-item Revised version.

g IES, Intuitive Eating Scale.

*** p < 0.001;

** p < 0.01;

* p < 0.05.

To assess the extent to which late eating accounted for a portion of these main effects, we examined whether the distributions of participants who reported late eating effects on dreams differed as a function of whether they thought that eating certain foods influenced their dreams. This analysis revealed that 26.5% (9/34) of participants who claimed that eating certain foods affected their dreams (category 5 in Table 1) also claimed that eating late affected their dreams, whereas only 9% (32/347) of participants who did not claim that eating specific types of foods affected their dreams did so (χ^2^ = 9.59, *p* < 0.006, Fisher exact test, 2-tailed). Thus, several participants who described specific, dream-altering foods also attributed dream changes to eating late at night.

Following this, we reproduced the previous MANCOVA comparisons while treating the effect of eating late on dreaming as a covariate. This changed the pattern of results in several respects. Most importantly, the **Dreams/Nightmares** multivariate group difference was no longer significant (*T* = 0.006, *F*_2, 327_ = 1.00, *p* = 0.369) and the groups no longer differed on the *disturbing dreams* univariable (*F*_1, 328_ = 1.57, *p* = 0.211). The **Sleep Disturbance** multivariate effect was also diminished but remained significant (*T* = 0.034, *F*_5, 335_ = 2.52, *p* = 0.029), as did univariate effects for *daytime dysfunction* (*F*_1, 339_ = 4.13, *p* = 0.043) and *difficulty falling asleep* (*F*_1, 339_ = 8.55, *p* = 0.004), but not for *sleep dissatisfaction* (*F*_1, 339_ = 0.32, *p* = 0.571) or *hours of sleep* (*F*_1, 339_ = 1.24, *p* = 0.267). For **Diet Quality**, the univariate difference for *drinking coffee* was eliminated (*F*_1, 330_ = 1.67, *p* = 0.199). For **Eating Motivation**, the multivariate effect was diminished (*T* = 0.021, *F*_4, 339_ = 1.96, *p* = 0.105), and the *reliance on hunger/satiety cues* difference was reduced to a trend (*F*_1, 342_ = 3.38, *p* = 0.067); the *emotional eating* difference, however, became significant (*F*_1, 342_ = 5.82, *p* = 0.016).

### Relationships between dreaming and other factors

To assess general relationships between the two dreaming factor scores and other variables, independent of whether participants reported food-dependent dreaming, we re-examined the correlations between *disturbing dreams* and *vivid dreams* and the other variables reported in Table 3 after covarying Age and FDD status. As shown in Table 5, *disturbing dreams* were associated with a largely pathological constellation of variables. There was a strong relationship with sleep disturbance as indicated by highly significant correlations with all four *SQS* subscales (all *p* < 0.00001) but not with hours of sleep. *Disturbing dreams* were also positively associated with being *currently on a diet* (*p* < 0.0004) and engaging in *binge-eating* (*p* < 0.00003), as well as with greater *cognitive restraint* (*p* < 0.004), more *uncontrolled eating* (*p* < 0.003), more *emotional eating* (*p* < 0.020), and less *reliance on hunger/satiety cues* (*p* < 0.005). *Disturbing dreams* were not, however, significantly related to the quality of participants' diets, having only a marginal positive correlation with *drinking coffee* (*p* = 0.097). To determine if these relationships with *disturbing dreams* might be due to a mediating effect of sleep disturbances, correlations were recalculated with the *SQS subscale* scores partialed out. The relationship of *disturbing dreams* to being *currently on a diet* was reduced to marginal significance (*p* = 0.070), while the relationship with *binge-eating* remained significant (*p* < 0.005). Among the motivational factors, however, only *cognitive restraint* remained significant (*p* < 0.012), while *emotional eating* (*p* = 0.276) and *reliance on hunger/satiety cues* (*p* = 0.111) became nonsignificant and *uncontrolled eating* was reduced to marginal significance (*p* = 0.090).

**Table 5:** **Pearson correlations between *Disturbing Dreams* and *Vivid Dreams* factor scores and sleep, diet, and eating measures for sample with Age and FDD status covaried (columns 2,3), and with sleep disturbance measures also covaried (columns 4,5)**.

	**Disturbing Dreams**	**Vivid Dreams**	**Disturbing Dreams**	**Vivid Dreams**
	**Partial r^a^**	**Partial r^a^**	**Partial r^b^**	**Partial r^c^**
**Sleep disturbance**				
SQS: daytime dysfunction	0.340^****^	−0.133^*^		
SQS: difficulty falling asleep	0.308^****^	−0.064		
SQS: sleep dissatisfaction	0.241^****^	−0.127^*^		
SQS: poor sleep maintenance	0.291^****^	0.055		
Hours of sleep	−0.023	0.104^*^		
**Diet quality**				
Healthy foods^b^	0.030	0.141^**^	0.056	0.137^**^
Unhealthy foods^c^	0.006	−0.158^**^	−0.034	−0.147^**^
Coffee	0.087^†^	0.067	0.015	0.093^†^
**Eating behavior**				
Currently on diet (1 = yes; 2 = no)	−0.185^***^	−0.043	−0.096^†^	−0.079
Longest time between meals	−0.005	0.188^***^	0.026	0.183^***^
Binge eating	0.217^***^	−0.070	0.151^**^	−0.041
**Eating motivation**				
TFEQ-18R: CR	0.155^**^	0.074	0.133^*^	0.080
TFEQ-18R: UE	0.156^**^	−0.164^**^	0.089^†^	−0.144^**^
TFEQ-18R: EE	0.122^*^	−0.080	0.058	−0.054
IES: Hunger/satiety cues	−0.147^**^	0.090^†^	−0.084	0.057

**** p < 0.00001;

*** p < 0.001;

** p < 0.01;

* p < 0.05;

† p < 0.10; Abbreviations as in Table 4.

a Covariates: Age, FDD status.

b Covariates: Age, FDD status, all SQS subscales.

c Covariates: Age, FDD status, SQS daytime dysfunction, SQS sleep dissatisfaction, Hours of sleep.

The *vivid dreams* factor showed a profile of correlations largely opposite to that for *disturbing dreams*, being associated with better sleep and more adaptive diet-related behaviors and motivations. More specifically, *vivid dreams* correlated negatively with *daytime dysfunction* (*p* < 0.012) and *sleep dissatisfaction* (*p* < 0.016) and positively with *hours of sleep* (*p* < 0.051). *Vivid dreams* were also associated with eating more *healthy foods* (*p* < 0.007) and fewer *unhealthy foods* (*p* < 0.003), and with longer *times between meals* (*p* < 0.0004). There was also a negative association with *uncontrolled eating* (*p* < 0.002) and a marginal positive association with *reliance on hunger/satiety cues* (*p* < 0.086). Covarying the relevant sleep measures (*SQS daytime dysfunction, SQS sleep dissatisfaction, and hours of sleep*) had little effect on the results for the remaining variables. The positive correlation with *healthy foods* (*p* < 0.009) and negative correlation with *unhealthy foods* (*p* < 0.006) remained, while a marginally positive correlation with *drinking coffee* (*p* < 0.077) emerged. The positive correlation with longer *times between* meals (*p* < 0.0006) and negative correlation with *uncontrolled* eating (*p* < 0.006) also remained; however, the marginally positive correlation with *reliance on hunger/satiety cues* was eliminated (*p* = 0.280).

## Discussion

Among a large sample of university undergraduates, we found evidence supporting two general ideas about how food influences dreams. First, results show that a substantial proportion of individuals (17.8%) perceive that food can render their dreams more bizarre or disturbing. These individuals are able to provide examples—some quite specific—of foods that they believe affect their dreams; as well, many of them perceive that eating late at night can affect their dreams. Second, we found evidence that disturbing dreams and vivid dreams are, in general, associated with several factors related to food consumption, such as different motivations for eating, with the effects for disturbing dreams being largely mediated by sleep quality. In both sets of results, we found evidence that degree of psychopathology vs. wellness might mediate the relationship between food and the types of dreams one has.

### Perceptions of food-dependent dreaming

Our finding that 16.7% of males reported food-dependent dreaming is surprisingly similar to the early estimate by Laird (Laird, 1920) that 15% of males believed dreaming can be affected by the type or amount of food eaten. Females were not studied by Laird but our estimate of 18.4% for females suggests that they do not differ substantially from males. It is noteworthy that our estimates of perceived food-dependent dreaming among university juniors are very similar to previous estimates of the prevalence of food and orality themes in the dreams of college students in both the US (16% males; 17% females; Hall and van de Castle, 1966) and Japan (22%; Yamanaka et al., 1982). These similarities raise the intriguing possibility that participants who report food-dependent dreaming are also those who dream most about food themes.

While we consider the present findings preliminary and in need of further replication and extension, there are several reasons to expect that our relatively modest prevalence estimates of perceived food-dependent dreaming are, in fact, underestimations. We queried participants about food in relation to only two common types of dreams, disturbing and bizarre, but details about other dream types or dream attributes could prove even more informative. Probes about sex dreams, flying dreams, lucid dreams, dreams of falling, or especially dreams about eating, drinking, and procuring or preparing food might well trigger other memories or attributions about the influence of foods. Further, the present sample was restricted to primarily young undergraduates who may have had less experience observing the influence of food on their dreams or have had fewer opportunities to be exposed to commonly held beliefs about relationships between food and dreaming. Consistent with this possibility, we found that FDD+ participants were on average 2.5 years older than FDD− participants. A more thorough evaluation of the many possible influences of food on dreams, especially with older participants, may well produce higher estimates of perceived food-dependent dreaming.

Although our exploratory use of open-ended responses for classifying foods led to varying levels of descriptive detail, and thus limited comparability among participants, some trends nevertheless emerged. Dairy products were the foods most frequently identified as influencing both disturbing (44%) and bizarre (39%) dreams. These proportions might be even higher in that some of the foods referred to by participants as “greasy” or “starchy” (e.g., pasta) may well have included cheese as a major ingredient. Sweet foods were also frequently mentioned as influencing dreams, but primarily in relation to bizarre (31%) rather than disturbing (13%) dreams. In contrast, spicy foods were predominantly mentioned as a precursor to disturbing dreams (19%) as were starchy foods (16%) and meat (13%). It is noteworthy that dairy and spicy foods were the two categories most often named as affecting disturbing dreams; together they constitute a striking parallel to the spiced cheese dish of Welsh rarebit. Cucumbers, which Jones (1931) described as the food most commonly believed to be a precursor of nightmares, was mentioned once within this category in the form of pickles. Eating late was also perceived to affect dreams—primarily disturbing dreams (47%) but also dreams that participants characterized as “bizarre,” “vivid,” “odd,” “strange,” or “crazy” (22%). A quarter of participants who perceived that specific foods affected their dreams also reported that eating later affected their dreams (which is likely an underestimation given that some of those who gave an example of late eating affecting a change in sleep may also have been able to give an example of a change in dreaming); this effect of late eating may reflect the possibility that specific foods eaten just before bed are those to which dream changes are most likely to be attributed. But it may also reflect the fact that eating late negatively impacts sleep (Crispim et al., 2011) which may, in turn, influence dreaming. Thus, whereas dairy products were seen as the most common instigators of both disturbing and bizarre dreams, other types of foods were mentioned as affecting predominantly either disturbing dreams (spicy, starchy, meat) or bizarre dreams (sweets), with late eating in many instances possibly mediating the perceived relationship between particular types of food and dreaming.

Comparisons of FDD+ and FDD− groups produced a set of differences consistent with the possibility that FDD+ is associated with higher levels of psychopathology which may in turn play a role in the tendency to perceive food-dependent dreaming. FDD+ participants reported having more disturbed sleep and disturbing dreams and, in a possibly related vein, drank more coffee. The FDD+ group was also somewhat more likely to eat for emotional reasons and was less sensitive to internal hunger and satiety cues. Tendencies toward emotional eating and low sensitivity to hunger/satiety cues are interrelated (van Strien et al., 2005)—as they were in the present study (*r* = −0.214, *p* < 0.0001)—and have been associated with other pathological symptoms. For example, high emotional eaters are characterized by eating more when distressed (van Strien et al., 2012) and have more blunted cortisol stress response (van Strien et al., 2013), poorer coping skills (Spoor et al., 2007), poorer interoceptive sensitivity (Herbert et al., 2013), and higher alexithymia and difficulty in identifying feelings (Larsen et al., 2006; van Strien and Ouwens, 2007). Emotional eating is also associated with posttraumatic stress disorder (PTSD), with degree of emotional eating being predictive of symptom severity (Talbot et al., 2013). Late eating may play a role in mediating some of these effects insofar as covarying the perceived effect of late eating on dreams somewhat diminished the differences in pathology between the two groups.

The observed relationship between FDD+ and higher psychopathology is not generally consistent with the findings of the British Cheese Board (2005), i.e., that eating cheese prior to sleep does not induce nightmares. While our findings do not demonstrate empirically that nightmares result from eating dairy, the fact that subjects themselves reported observing this phenomenon, as well as our finding that such observations are correlated with psychopathological measures, together tend to support the opposite conclusion: that eating cheese may, in some individuals, adversely influence subsequent dreaming.

Although the present results for perceived food-dependent dreaming are clearly limited by their correlational nature and by our use of subjective and retrospective reports, and should therefore be considered as exploratory in nature, we nonetheless suggest that they provide sufficient grounds for considering four possible explanations for why changes in dreams are often attributed to foods. Because our results are preliminary, we frame these explanations in the form of hypotheses for future study.

#### Food specificity hypothesis

According to this hypothesis, many participants who report food-dependent dreaming are accurately describing cause-and-effect relationships between specific foods or food categories and the content or quality of their dreams. In this context, FDD+ participants may differ from FDD− participants simply in having a greater “dream sensitivity” to certain foods or in being more accurate observers of food-dream relationships. The purported results of the aforementioned unpublished study sponsored by the British Cheese Board (2005)—which claimed, for example, that eating cheddar cheese led to dreams about celebrities—would, if valid, constitute an extreme example of this type of effect. The British Cheese Board report noted that cheese contains high levels of tryptophan which has been shown to reduce stress and promote sleep. But although this might account for a tendency for cheese to induce more pleasant or relaxing dreams, which the present results would dispute, it seems insufficient to account for a certain type of cheese (e.g., cheddar) leading to a specific change in dream content (e.g., celebrities). Nevertheless, as previously noted, several foods contain nutrients that have been shown to impact sleep, and it is possible that certain foods could potentially also impact dreaming—though one suspects such effects would be of a more general nature (e.g., calm vs. exciting dreams) than of a specific change in dream content.

#### Food distress hypothesis

Closely related to the above, this hypothesis proposes that the perception of food-dependent dreaming may result from nonspecific effects of food intolerances and other adverse reactions to food, the symptoms of which result in bizarre and disturbing dreams. As noted earlier, this notion dates back to antiquity, with Hippocrates himself believing it to be a significant cause of nightmares. It differs from the food specificity hypothesis in proposing that the effect on dreams is not the result of a specific link between a particular food or type of food (or food nutrient) and dreaming; rather, the effect is the result of an adverse change in one's somatic condition, especially in the alimentary system, which could potentially also have been induced by a non-food event such as illness or other stressor. The adverse change in condition could either directly impact dreaming or indirectly affect dreaming through the disruption of sleep patterns (Bohn et al., 2013; Dominguez-Ortega et al., 2014). In fact, sleep disruption is correlated with a number of food items commonly known to induce gastrointestinal symptoms (Bohn et al., 2013). Among the more common are dairy products (e.g., 49% of patients with irritable bowel syndrome), beans/lentils, chocolate, and wheat (Benton, 2007; Bohn et al., 2013). The fact that dairy products were by far the most frequently named instigator of both bizarre and disturbing dreams in the present study supports this hypothesis, especially in view of the relatively high prevalence of lactose intolerance among Canadians (20% of females and 12.3% of males; Barr, 2013). Also consistent with this possibility, many of the physical symptoms of lactose intolerance (gas, bloating, cramps, diarrhea) were reported spontaneously in response to our open-ended question about how eating late at night influences sleep and dreams.

Spicy foods were also singled out in our study as influencing dreams, particularly disturbing dreams (18.8%). Some pungent spices are known to affect sleep and might thus also alter dreaming. For example, a meal containing a combination of Tabasco sauce and hot English mustard caused healthy male athletes to sleep fewer hours and less efficiently (e.g., more time awake) than did a meal without these spices (Edwards et al., 1992). Moreover, rectal temperature was significantly higher during the first REM period of the night following the spiced meal, a change that might directly alter dream formation processes since REM sleep is the stage most conducive to copious, vivid dream content.

In sum, food sensitivities and intolerances, especially lactose intolerance, could play a significant role in food-dependent dreaming. Assessment of food allergies, sensitivities, and intolerances in future work should clarify this potential explanation.

#### Folklore hypothesis

This hypothesis stipulates that an individual's tendency to perceive particular foods as affecting their dreams originates in the assimilation of beliefs about food that have been transmitted inter-generationally within families, groups, or the broader culture. While not incompatible with the previous hypotheses—a cultural belief that, e.g., spicy foods can lead to bad dreams may in fact be accurate—the *folklore hypothesis* does not depend upon an individual's accurate assessment of a food-dream relationship. That is, some members of a culture may falsely believe that a prevalent folktale about, say, pickles causing bizarre dreams applies to them when it does not. Thus, our finding that dairy products are the most frequently mentioned type of food deemed to be responsible for inducing disturbing (43.7%) and bizarre (38.5%) dreams, while plausible from a lactose intolerance perspective, may also be consistent with the folklore hypothesis. As previously mentioned, there appears to be a belief prevalent in certain populations that dairy products can affect dreams; this belief dates back at least to the early 1900s, as exemplified by McCay's *Dream of the Rarebit Fiend* cartoon series. While it is doubtful that contemporary university students are familiar with the *Rarebit Fiend* series, its wide influence at the time (see review in Wikipedia, 2014, http://en.wikipedia.org/wiki/Dream_of_the_Rarebit_Fiend) may have ensured that its central message (“eating spicy cheese leads to bizarre dreams”) would continue in common folklore for generations. As earlier noted, the persistent prevalence of this belief in Britain, whatever its source, appears to have been the motivation behind the unpublished study on the effect of cheese on dreams by the British Cheese Board (2005).

A related notion is that cultural beliefs about the effects of particular foods may interact with the ingestion of those foods such that the interaction brings about the culturally sanctioned belief. Thus, eating a lot of cheese before going to bed while being reminded that this will surely cause bizarre dreams may enhance the likelihood that bizarre dreams will occur.

#### Misattribution hypothesis

This hypothesis proposes that some perceived linkages between food and dreams may be false, with the connection to food, or a particular food, being a misattribution of the real cause. As shown in the preceding section, folklore may have a particularly strong influence on this type of misattribution. For example, students who are taught to believe that cheese can induce nightmares may be highly prone to attributing bad dreams to a pizza they ate the night before a stressful exam rather than to their worries about the exam. Interestingly, FDD+ participants may be especially prone to misattributing the cause of a dream in this way if their low reliance on hunger/satiety cues is representative of a more general lack of internal awareness which then leads them to seek an external explanation for their dreams.

As should be clear from the above discussion, these four explanations for the perception of food-dependent dreaming are not mutually exclusive. Any particular instance of a perceived food-dream relationship may result from a combination of two or more of these factors.

### General relationships between food and dreaming

Independent of participants' beliefs in food-dependent dreaming, the present study also found evidence of at least two relationships between food and dreaming. The first relationship, which was also noted for the perception of food-dependent dreaming, reflects a pathological constellation of variables that are uniquely associated with disturbing dreams. In addition to disordered sleep, these variables include being on a diet, binge-eating, cognitive restraint, uncontrolled eating, emotional eating, and reduced reliance on hunger/satiety cues. The association of these variables with disturbing dreams could not be attributed uniquely to disordered sleep because correlations with the other measures persisted even when disordered sleep was covaried.

In contrast to the above, the second type of relationship between food and dreaming reflects a constellation of wellness measures uniquely associated with vivid dreams. These measures include better sleep, a healthier diet, longer daily intervals without eating, and less uncontrolled eating. Again, the relationships held even when the sleep measures were covaried, thus excluding better sleep as a unique explanation for the relationship.

These two opposing patterns of correlations partially replicate the preliminary findings of Kroth et al. (2007). Whereas the latter authors reported that dream recall, recurrence, pleasantness and incidence of several specific themes, such as sexuality, risk-taking and flying, were all positively correlated with the frequency of eating organic foods, we found a positive correlation between a more general measure of dream vividness that overlaps conceptually with their measures (dream recall especially) and a measure of eating healthy foods. Similarly, whereas Kroth et al. found a negative correlation between dream recall and a preference for eating junk foods, we too found a negative correlation between vivid dreams and eating unhealthy foods. One finding reported by Kroth et al. that we failed to replicate was a negative correlation between nightmares and a preference for eating junk foods; that is, they found that less frequent nightmares were associated with a preference for unhealthy foods, whereas we found no association between disturbing dreams and diet quality.

The present findings further demonstrate relationships between dietary patterns and disturbing and vivid dreams. In particular, cognitive restraint, emotional eating, and reduced reliance on hunger/satiety cues were associated with disturbing dreams whereas uncontrolled eating was associated with both disturbing dreams (higher scores) and vivid dreams (lower scores). At least two of these measures, emotional eating and reliance on hunger/satiety cues, may reflect individual differences in visceral or affective awareness. If this is the case, these findings, in addition to the general findings about food-dependent dreaming, could be relevant to an understanding of how consciousness in the form of interoceptive awareness may contribute to dream formation. For example, alexithymia, and the alexithymia component factor *difficulty in identifying feelings*, are both elevated in several types of eating disorders (Troop et al., 1995), as well as in subjects who report more disturbing dreams (Nielsen et al., 2011). This, and the fact that individuals with eating disorders frequently report disturbing dreams, e.g., increased ineffectuality, feelings of impending doom, hostility, anxiety, anger (Brink and Allan, 1992; for review see Lauer and Krieg, 2004), suggests that a chronic inability to access interoceptive sensations may be a factor common to both eating disorders and disturbing dreams. This further suggests that therapeutic approaches that promote a generalized awareness and identification of feelings—which may include feelings within one's dreams (e.g., Gendlin, 1986)—may be particularly helpful in the treatment of both disturbed dreaming and eating disorders.

Particularly intriguing is our finding that participants who reported experiencing longer daily intervals without eating also reported more vivid dreams. As noted earlier, fasting has been utilized since ancient times as a means of inducing vivid dreams. The present results suggest that even brief periods of food deprivation within a day may be sufficient to produce a similar effect. This possibility is interesting in view of growing research indicating that intermittent fasting—consisting of periodic intervals of 12–24 h with little or no food—can have beneficial effects on both physical health and cognitive abilities (e.g., Longo and Mattson, 2014). These findings are especially relevant if the longer intervals without eating reported in the present study typically occurred either following sleep (resulting in a skipped breakfast) and/or preceding sleep (after an early supper), in which case these participants may have regularly experienced extended periods of food deprivation consistent with an intermittent fasting protocol. If so, then the relationship of food deprivation with vivid dreams found in the present study may be associated with, or the result of, a general improvement in cognitive functioning resulting from exposure to periods of intermittent fasting. (See also Zilberter and Zilberter, 2013, for a discussion of empirical evidence concerning the benefits vs. adverse effects of skipping breakfast). This possibility is further supported by the fact that our scores on vivid dreams and longest time between meals were both positively correlated with scores on the Attentional Function Index (*r* = 0.181, *p* = 0.001; *r* = 0.157, *p* = 0.004, respectively), a standardized self-report measure of cognitive functioning (Cimprich et al., 2011) that was included in the survey as part of a questionnaire set for a different study. If replicated, this relationship between brief periods of fasting and vivid dreams might be useful in helping individuals to procure vivid dreams for use in creative pursuits or psychotherapy. It is noteworthy, however, that intervals of fasting may be confounded by a tendency to sleep late in the morning; “sleeping-in” may facilitate more vivid dreams because it takes place closer to the peak of the circadian REM sleep propensity rhythm (for review see Nielsen, 2011).

It is perhaps ironic that one of the earliest theories of disturbing dreams—that it is often the result of foods we have eaten—has generated such little empirical research. Moreover, when the possibility has been considered, it has often been in the form of what Jones (1931) aptly referred to as a “half-jocular remark concerning the assimilable capacity of the evening meal” (p. 13). The impact of food on dreams may, however, be deserving of more serious consideration. For example, as previously noted, PTSD has been found to be associated with emotional eating (Talbot et al., 2013); in view of the present results, this might mean that the disordered eating patterns associated with PTSD play a role in exacerbating or maintaining the chronic nightmares that constitute a major symptom of PTSD. This in turn suggests that targeting the development of healthier patterns of eating may be a useful adjunct in the treatment of PTSD. The present results also suggest that dreams, and perceived food-dream relationships, may be useful indicators of food intolerances and maladaptive patterns of eating.

Further research on the topic seems warranted. This should include additional survey research using a more refined set of items as informed by the present findings, as well as experimental research which directly manipulates foods eaten and assesses their effects on dreaming. Of interest would be assessments of the extent to which certain foods—especially those, like Welsh rarebit, that combine dairy and spiciness—and particular eating patterns play a role in the exacerbation of chronic nightmares, as well as of the relationship of vivid dreams to intermittent periods of food deprivation and improvements in cognitive functioning. Such studies may even cast light on how a seeming relic from cartoon history, the *Dream of the Rarebit Fiend*, achieved such popularity and continues to portray a common belief about food and dreams today.

### Conflict of interest statement

The authors declare that the research was conducted in the absence of any commercial or financial relationships that could be construed as a potential conflict of interest.
