# Supplementary material for: Dreams of the Rarebit Fiend: food and diet as instigators of bizarre and disturbing dreams
Source: Front Psychol. 2015 Feb 17;6:47. doi: 10.3389/fpsyg.2015.00047 (PMC4330685; doi:10.3389/fpsyg.2015.00047)
Supplement: Supplementary file 1 [file DataSheet1.DOCX]

**Supplemental Material**

**Appendix: In-house survey items**

| **Item** | **Response choices** |
| --- | --- |
| **Dream characteristics** |  |
| 1. A disturbing dream is any dream that contains very negative or unpleasant emotions. How many disturbing dreams did you recall during the past two weeks? | a. None  b. 1-2  c. 3-4  d. 5-6  e. 7-8  f. 9-10  g. 11-12  h. 13-14  i. 15 or more (that is, on average, more than one per night) |
| 2. How distressed were you by these disturbing dreams? | Not at all Moderately Extremely  1 2 3 4 5 6 7 |
| 3. In general, what percentage of these disturbing dreams were so bothersome that you were forced to wake up (as opposed to just naturally waking up at that time)? | a. 0%  b. 10%  c. 20%  d. 30%  e. 40%  f. 50%  g. 60%  h. 70%  1. 80%  J. 90%  k. 100% |
| 4. How many dreams that were NOT disturbing did you recall over the past two weeks? | a. None  b. 1-2  c. 3-4  d. 5-6  e. 7-8  f. 9-10  g. 11-12  h. 13-14  i. 15 or more (that is, on average, more than one per night) |
| 5. In general, how clearly were you able to recall your non-disturbing dreams from the past two weeks? | Not at all Very  1 2 3 4 5 6 7 |
| 6. In general, how realistic were your non-disturbing dreams from the past two weeks (that is, your dreams made sense even if some of the elements were unusual or impossible, such as having an ability to fly)? | Not at all Very  1 2 3 4 5 6 7 |
| 7. How often were these dreams in colour? | Never Half the time Always  1 2 3 4 5 6 7 |
|  |  |
| **Perception of food-dependent dreaming** |  |
| 1. Have you ever noticed certain foods that seemed to lead you to have a disturbing dream? | a. No b. Yes |
| 2. What foods have you noticed that seemed to lead you to have a disturbing dream (please be as specific as possible)? | Open-ended |
| 3. Have you ever noticed certain foods that seemed to lead you to have a bizarre dream? | a. No b. Yes |
| 4. What specific foods have you noticed that seemed to lead to bizarre dreams? | Open-ended |
| 5. Have you ever noticed that eating late at night seems to affect your dreams or your sleep? | a. No b. Yes |
| 6. In what way does eating late at night affect your dreams or your sleep (please be as specific as possible)? | Open-ended |

| **Sleep length** |  |
| --- | --- |
| On average, how many hours do you sleep per day (including sleeping that occurs both at night and during the day)? | a) 3 hrs or less  b) 4 hrs  c) 5 hrs  d) 6 hrs  e) 7 hrs  f) 8 hrs  g) 9 hrs  h) 10 hrs  i) 11 hrs or more |
| **Diet quality** |  |
| 1. How often do you eat green salad (with or without other vegetables)? | 1 = Never  2 = 1 to 3 times per month  3 = 1 to 2 times per week  4 = 3 to 4 times per week  5 = 5 to 6 times per week  6 = 1 time per day  7 = 2 times per day  8 = 3 times per day  9 = 4 times per day  10 = 5 or more times per day |
| 2. How often do you eat vegetables (raw, cooked, canned or frozen), NOT COUNTING potatoes and salad? | same |
| 3. How often do you eat fruit (raw, cooked, canned or frozen), including berries? | same |
| 4. How often do you eat whole grain foods, such as whole grain bread or pasta, brown rice, oatmeal porridge, or wheaties? | same |
| 5. How often do you consume NON-diet soft drinks (e.g., Coke, Pepsi, Dr. Pepper, etc.)? | same |
| 6. How often do you eat low nutrient snacks like cookies, potato chips, and candy bars? | same |
| 7. How often do you drink regular coffee (coffee that contains caffeine)? | same |
| **Eating Behavior** |  |
| 1. Are you presently on a calorie restricting diet to lose weight? | A = yes; B = no |
| 2. During a typical day, from when you get up in the morning to when you go to bed, what is the longest amount of time that passes during which you do not eat anything or have any calorie-containing drink (such as a non-diet soft drink or coffee with cream and/or sugar)? | a. less than 1 hour  b. 1 to 2 hours  c. 2 to 3 hours  d. 3 to 4 hours  e. 4 or more hours |
| 3. How many DAYS per week on average over the past 6 MONTHS have you eaten an unusually large amount of food and experienced a loss of control (you feel that you couldn’t stop eating or control what or how much you were eating)? | # days/week:  0 1 2 3 4 5 6 7 |
